# Efficient FLP-mediated germ-line recombination in *C. elegans *

**DOI:** 10.17912/W2G66S

**Published:** 2018-03-19

**Authors:** Javier Macías-León, Peter Askjaer

**Affiliations:** 1 Andalusian Center for Developmental Biology (CABD), CSIC/JA/Universidad Pablo de Olavide, 41013 Seville, Spain

**Figure 1:**
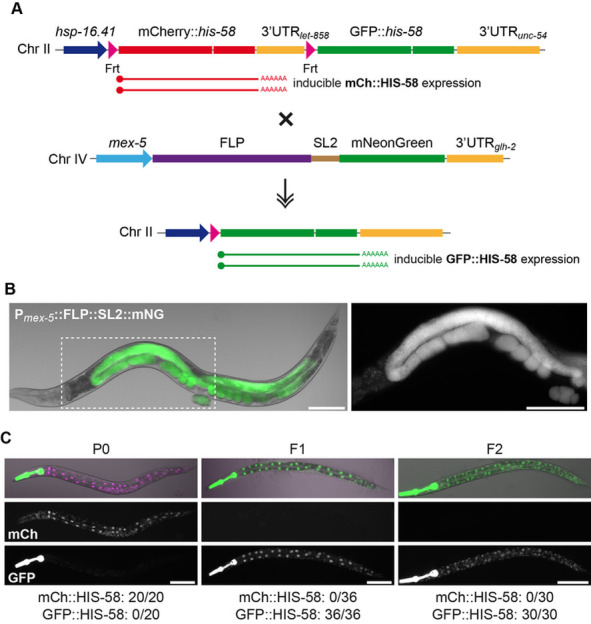
(A) Schematic representation of transgenes before and after recombination. (B) The P*mex-5*::FLP::SL2::mNG transgene is exclusively expressed in the germ line and early embryos. Boxed area is shown enlarged on the right. (C) Induction of the dual color reporter produces red nuclei only prior to recombination (P0; left panel), whereas ubiquitous GFP::HIS-58 expression is observed after recombination (F1 and F2; middle and right panel). Pharyngeal GFP expression was used to facilitate the cross and is independent of FLP-mediated recombination. Numbers below indicate animals analyzed. Scale bars 0.1 um.

## Description

**​**Recombinases are very useful enzymes for spatiotemporal control of gene expression (Hubbard 2014). Through binding to specific DNA sequences, they can be used to excise or exchange DNA fragments. We have previously generated a series of *Caenorhabditis elegans* strains that stably express an efficient FLP recombinase in specific tissues and reported how they enable precise regulation of gene expression, cell ablation or conditional gene knockout (Muñoz-Jiménez et al 2017). However, these strains are restricted to somatic tissues. Here we describe a novel strain that expresses FLP specifically in the germ line from an integrated single-copy transgene. Employing a dual color reporter, we found ~100% recombination efficiency, implying that the strain can be useful for a variety of experiments. This includes 1) induce germ-line expression of genes that have a negative effect on animal fitness (and thus are difficult or impossible to maintain as stably expressing strain); 2) permanently remove selectable markers flanked by Frt sites by easy crossing. For comparison, the elegant SapTrap toolkit for CRISPR/Cas9 genome modification relies on a loxP-flanked *unc-119(+)* transgene, whose removal requires injection of a Cre expression plasmid (Schwartz & Jorgensen 2016); 3) stable and precise knockout (KO) of genes flanked by Frt sites or tagged by a unique GFP KO cassette (Muñoz-Jiménez et al 2017).

To generate plasmid pBN396 for germ-line expression of FLP, we inserted a *Not*I+*Kpn*I fragment from pBN179 P*mex-5*::*gfp*::*mel-28* (Gómez-Saldivar et al 2016) into pBN338 P*dat-1*::FLP::SL2::mNG (Muñoz-Jiménez et al 2017) cut with the same enzymes. This fragment contains 486 base pairs immediately upstream of the *mex-5* start codon (5’- atatcagtttttaaaaaatt…ttgaatgtttcagacagaga-3’).

Plasmid pBN396 contains an SL2 transplicing cassette between FLP and mNeonGreen (mNG), thus transcription from the *mex-5* promoter produces a bi-cistronic pre-mRNA that encodes individual FLP and mNG proteins (Figure 1A). After sequence verification we injected pBN396 into EG6700 *unc-119(ed3)* III; *cxTi10882* IV together with relevant plasmids for Mos1-mediated single-copy integration (Frøkjær-Jensen et al 2012), producing strain BN711 *unc-119(ed3)* III; *bqSi711*[pBN396(*unc-119(+)* P*mex-5*::FLP::SL2::mNG)] IV. We demonstrated by PCR that the Mos1 transposon was not re-inserted elsewhere in the genome of BN711. Correct expression of FLP in the germ line was indirectly confirmed by co-expression of fluorescent mNG (Figure 1B). To evaluate recombination efficiency, we next crossed BN711 hermaphrodites with BN596 males, which carry a single-copy dual color reporter inserted in chromosome II and a pharyngeal GFP marker in the *cxTi10882* locus on chromosome IV (Muñoz-Jiménez et al 2017; Figure 1A). F1 progeny were incubated at 34°C for 15 min followed by recovery at 20°C for 3 h and evaluated in a fluorescent stereoscope. All animals (n=36) had ubiquitous expression of GFP::HIS-58, whereas no expression of mCherry::HIS-58 was observed (Figure 1C middle panel). This suggested that the FLP enzyme had effectively induced recombination in all blastomers of the early embryo. We next analyzed F2 progeny produced by F1 self-fertilization and found again ubiquitous GFP::HIS-58 expression after heat induction, even in animals that did not inherit the FLP transgene (n=30; Figure 1C right panel). Together, this demonstrated that the FLP transgene efficiently induces germ-line recombination, which is stably propagated to subsequent generations.

In conclusion, we have developed an efficient addition to the FLP/Frt toolkit for *C. elegans*, enabling manipulation of gene expression in the germ line. The novel FLP plasmid and FLP line will be made available to the community trough Addgene (https://www.addgene.org) and the *Caenorhabditis* Genetics Center (https://cgc.umn.edu), respectively.

## Reagents

Plasmid pBN396 (*unc-119(+)* P*mex-5*::FLP::SL2::mNG). Strain BN596 *bqSi294*[pBN154(*unc-119(+)* P*hsp-16.41*::FRT::*mCh*::*his-58*::FRT::*gfp*::*his-58*)] II; *bqSi577*[pBN306(*unc-119(+)* P*myo-2*::*gfp*)] IV. Strain BN711 *unc-119(ed3)* III; *bqSi711*[pBN396(*unc-119(+)* P*mex-5*::FLP::SL2::mNG)] IV.
